# The Comparison of the Effect of Childbirth Preparation Classes and Spirituality‐Based Counseling on Childbirth Fear Among Nulliparous Pregnant Women: A Quasi‐Experimental Study

**DOI:** 10.1002/hsr2.71879

**Published:** 2026-02-22

**Authors:** Fatemeh Golnazari, Sousan Heydarpour, Aliakbar Foroughi, Fatemeh Heydarpour

**Affiliations:** ^1^ Student Research Committee School of Nursing and Midwifery Kermanshah University of Medical Sciences Shahid‐Beheshti Kermanshah Kermanshah Iran; ^2^ Department of Reproductive Health School of Nursing and Midwifery Kermanshah University of Medical Sciences Kermanshah Iran; ^3^ Department of Clinical Psychology School of Medicine Kermanshah University of Medical Sciences Kermanshah Iran; ^4^ Medical Biology Research Center, Health Technology Institute Kermanshah University of Medical Sciences Kermanshah Iran

**Keywords:** childbirth preparation classes, fear of childbirth, primiparous women, spirituality‐based counseling

## Abstract

**Background and Aims:**

Fear of childbirth is a common issue during pregnancy. This study aimed to compare the effects of childbirth preparation classes and spirituality‐based counseling on the levels of childbirth fear in primiparous pregnant women.

**Methods:**

This quasi‐experimental study involved 90 primiparous pregnant women. Participants were randomly selected and were subsequently divided into three groups: (A) childbirth preparation classes (*n* = 30), (B) spirituality‐based counseling (*n* = 30), and (C) a control group (*n* = 30). Group A participated in 8 weekly sessions of childbirth preparation classes in addition to routine care. Group B attended 8 weekly sessions of spirituality‐based counseling in addition to routine care, and Group C received only routine care. Three groups completed the delivery expectancy/experience questionnaire before, 1 week after, and 5 weeks after the intervention.

**Results:**

Before the intervention, the mean scores of fear of childbirth were 50.97 ± 4.75, 53.23 ± 4.87, and 53.03 ± 5.12 in the A, B, and C groups, respectively, and there were no statistically significant differences in the mean scores of fear of childbirth among the three groups. One week after the intervention, the mean scores for fear of childbirth were significantly lower in the A and the B groups compared with the C group (40.20 ± 4.97 and 43.06 ± 10.14 vs. 54.66 ± 5.60, *p* < 0.001) and no significant statistical difference was seen between the A and B groups. Five weeks after the intervention, the mean scores for fear of childbirth were significantly lower in the A and the B groups compared with the C group (35.26 ± 4.20, 30.50 ± 6.13, *p* < 0.001), and a significant statistical difference was seen between group A and B (*p* = 0.001) with a greater reduction in group B.

**Conclusion:**

Both spirituality‐based counseling and childbirth preparation classes reduced levels of fear of childbirth in pregnant women.

## 1 Background

The fear of childbirth (FOC) is defined as severe anxiety that disrupts the daily functioning and well‐being of women [1, 2]. This issue is particularly prevalent among primiparous women due to their unfamiliarity with the childbirth process, with the primary cause being the fear of labor pain [3]. The prevalence of FOC varies across different countries and cultures [4]. For instance, it is approximately 22% in Scandinavian countries, the United Kingdom, Australia, and Sweden, while in Iran, it ranges from 17.3% to 89.3% [5, 6]. The FOC leads to an increased incidence of preterm birth and prolonged labor due to elevated catecholamine levels and increased uterine contractions. It also results in adverse fetal and neonatal outcomes, such as irregular fetal heart patterns, low birth weight due to increased uterine artery resistance from maternal anxiety, higher mortality rates at birth, and lower Apgar scores [1, 7]. FOC is a common reason for the rising demand for cesarean sections among primiparous women [8, 9].

According to studies, one effective strategy to reduce the FOC is participation in childbirth preparation classes during pregnancy. These classes provide psychological and educational interventions that positively impact the mental health of women during pregnancy and postpartum. The education provided in these classes offers an opportunity to correct misconceptions about childbirth, which contribute to fear, anxiety, reduced self‐efficacy, and low self‐esteem among women [10]. Additionally, childbirth preparation classes are currently part of the Health Transformation Plan in Iran, aimed at encouraging natural childbirth. In these classes, pregnant women receive education on childbirth and prenatal care, nutrition and exercise during pregnancy and lactation, proper breastfeeding techniques, and postpartum care [11].

A study demonstrated that childbirth preparation classes reduced the FOC [12]. A study indicated that participation in childbirth preparation classes had an effect on reducing maternal anxiety symptoms but did not impact the level of childbirth fear [13]. Mehrabadi et al. found that these classes increased the FOC, suggesting that the content of these educational programs needs to be reviewed [14].

Since the late 20th century, there has been a growing recognition of the existential dimension of human experience and suffering within clinical practice, including increased attention to patients' religious and spiritual worldviews [15, 16]. In response to this development, the American Psychological Association (APA) has established guidelines for addressing cultural, religious, and spiritual issues in therapeutic settings [17]. Furthermore, empirical research has highlighted the significance of spirituality in clinical contexts, demonstrating, for example, its protective effects on mental health [18].

Spiritual counseling prioritizes supporting mothers according to their beliefs, fostering spiritual support and hope to enable them to utilize spiritual coping mechanisms for reducing anxiety and fear during pregnancy and childbirth. Through this form of counseling, misconceptions and worries concerning pregnancy can be evaluated, pinpointed, and rectified, fostering greater trust among pregnant mothers. This approach facilitates enhanced empathy and the utilization of accessible resources to aid pregnant women [19]. A study showed that eight sessions of group counseling with a spirituality‐centered approach alongside childbirth preparation classes for 4 weeks (intervention group), compared to receiving routine prenatal care (control group), led to further improvement in adaptive patterns among primiparous pregnant women facing pregnancy challenges [20].

On the other hand, one study found no association between spiritual intelligence and FOC [21]. Meanwhile, other researchers have suggested that large‐scale intervention studies are needed to understand the impact of spirituality on FOC better [22]. Although childbirth preparation classes are common in Iran, there is no consensus on their effectiveness in reducing the level of childbirth fear. Given the prominent role of spirituality and spiritual well‐being in the field of health, the deep roots of spirituality in Iranian culture, and its pervasive influence on all aspects of Iranian society, along with the lack of similar national studies, conducting research in this area appears essential.

Furthermore, non‐pharmacological methods, which are free from adverse effects on both mother and fetus, are preferred by patients. Comparing widely practiced methods, such as childbirth preparation classes with other interventions, such as spirituality‐based counseling, can provide valuable insights for health policymakers regarding the effectiveness of these programs in promoting maternal health. Therefore, the present study was conducted to examine and compare the impact of childbirth preparation classes and spirituality‐based counseling on the FOC in primiparous women. We hypothesized that pregnant women who receive spirituality‐based counseling will have a lower degree of FOC than those in a childbirth preparation classes group.

## 2 Methods

### 2.1 Study Setting and Design

This quasi‐experimental study was conducted in health centers in Kermanshah, located in the western region of Iran, from November 2022 to May 2023. This study included two intervention groups—one receiving childbirth preparation courses and the other receiving spirituality‐based counseling—along with a control group. A total of 90 pregnant women participated in the study (*n* = 30 per group). Participants were randomly selected from the health centers using a random number table and divided into three groups without randomization. While randomization was not feasible due to logistical and ethical constraints in our clinical setting, we employed careful matching of participants across groups based on key demographic variables to minimize potential selection bias.

### 2.2 Sample Size Calculation

The sample size for each group was determined based on the findings of a previous similar study [14]. The following formula was used for the calculation:

$$n=\frac{{\left(Z_{1-\frac{\alpha }{2}}+Z_{1-\beta }\right)}^{2}(\delta_{1}^{2}+\delta_{2}^{2})}{{\left(\mu_{1}-\mu_{2}\right)}^{2}}$$

In this equation, the parameters were set as follows: *α* = 0.05, *β* = 0.1, *μ*₁ = 112.8, *μ*₂ = 86.7, *δ*₁ = 28.6, and *δ*₂ = 30.4, resulting in an initial sample size of *n* = 27 per group. Based on expert recommendations and to enhance the statistical power of the study, a type II error rate (*β*) of 0.1 was adopted, corresponding to a study power of 90%. To account for potential attrition, an additional 10% was added, leading to a final sample size of 30 participants per group.

### 2.3 Participants

Ninety pregnant women were recruited for the study. Eligibility for potential inclusion was determined using the Wijma Delivery Expectancy/Experience Questionnaire Version A (W‐DEQ‐A), with a score ranging from 38 to 84 indicating a moderate level of FOC. Screening was conducted through face‐to‐face interviews by a third party using the W‐DEQ‐A. The individual responsible for data collection was blinded to group allocation and received prior training to ensure consistency and reliability in data collection procedures.

The inclusion criteria were as follows: primiparous women with a singleton pregnancy, aged between 18 and 40 years, a gestational age of 20–24 weeks, willingness to participate in the study, a minimum of primary education, and a W‐DEQ‐A score between 38 and 84.

Exclusion criteria included withdrawal of consent or dissatisfaction with continued participation, the development of a high‐risk pregnancy, the onset or diagnosis of mental health disorders, the occurrence of adverse events during the intervention period, substance abuse (including alcohol or drug addiction), current use of anti‐anxiety or antidepressant medications, known mental or physical illnesses, absence from three or more intervention sessions, and any contraindications to physical activity during pregnancy as outlined by the American College of Obstetricians and Gynecologists (ACOG) guidelines.

The control group received standard prenatal care, which included assessments such as weight measurement, blood pressure monitoring, fundal height evaluation, fetal heart rate monitoring, as well as education on oral and dental health, screening for domestic violence, substance use (including smoking, alcohol, and drug use), and sexual health. This routine care was provided in accordance with the national guidelines established by the Iranian Ministry of Health and Medical Education and was uniformly administered across all three study groups.

In addition to receiving routine prenatal care, the group participating in childbirth preparation classes attended eight structured sessions conducted by two trained midwives. The content and delivery of these sessions adhered to protocols established by the Iranian Ministry of Health and Medical Education. The intervention was implemented in accordance with the national guidelines for childbirth preparation classes [23].

The eight childbirth preparation sessions covered the following topics: (1) objectives and principles of childbirth preparation classes; (2) nutrition during pregnancy; (3) mental health during pregnancy; (4) warning signs and the differentiation between normal and abnormal pain; (5) birth planning; (6) understanding the labor process, including its signs and stages; (7) postpartum health and care, including recognition of postpartum warning signs; and (8) infant health (Table 1).

**TABLE 1:** Content of preparedness classes for childbirth (according to the Guidelines of the Iran Ministry of Health and Medical Education).

“The sessions”	Time/gestational age in weeks	The topics
First	20–23	Explanation of objectives and principles of preparedness classes for childbirth, class schedule and dates, introduction to mothers, anatomical and physiological changes during pregnancy, personal hygiene, adaptation to pregnancy changes, and postural correction during pregnancy.
		– Engagement in skeletal muscle exercises, respiratory exercises, and relaxation techniques.
Second	24–27	Nutrition during pregnancy with emphasis on dietary intake and explanation of food pyramid, and postural correction during pregnancy.
		– Engagement in skeletal muscle exercises, respiratory exercises, and relaxation techniques.
Third	28–29	Psychological well‐being, spousal role, mood changes during pregnancy, fetal growth and development, communication with the fetus, and postural correction during pregnancy.
		– Engagement in skeletal muscle exercises, respiratory exercises, and relaxation techniques.
Fourth	30–31	Warning signs and diagnosis of normal and abnormal pregnancy pains, bleeding, spotting, headaches, fetal movements, rupture of membranes, edema of hands and feet, and postural correction during pregnancy.
		– Engagement in skeletal muscle exercises, respiratory exercises, and relaxation techniques.
Fifth	32–33	– Birth planning, selection of delivery method, types of pain management techniques, pain reduction during labor, choice of birthing location, necessary equipment for delivery, hospital labor room visit if possible, and postural correction during pregnancy.
		– Engagement in skeletal muscle exercises, respiratory exercises, and relaxation techniques.
Sixth	34–35	– Familiarization with the labor process and signs of labor pain, understanding the stages of labor, self‐care practices in each stage, role of pregnancy hormones in labor stages, coping mechanisms for labor pain, screening of childbirth films or animations, and postural correction during pregnancy.
		– Engagement in skeletal muscle exercises, respiratory exercises, and relaxation techniques.
Seventh	36	Postpartum health and care, postpartum warning signs, postpartum depression, breastfeeding education, and postpartum exercises.
Eighth	37	The emphasis on neonatal health, highlighting the general characteristics of newborns at birth, infantile period, infant care practices, bathing and diaper changing procedures, breastfeeding education, recognition of neonatal warning signs, presentation of breastfeeding and infant care films, in addition to postural correction during pregnancy.
		– Engagement in skeletal muscle exercises, respiratory exercises, and relaxation techniques.

Spirituality‐based counseling consisting of eight sessions was conducted once a week for 45–60 min each, held at healthcare centers in groups of 10–12 participants. The content of spiritual education was based on psychological–spiritual interventions proposed by Richard and Bergin (2005), as well as communication skills training from the spiritual education package by Ghahari, with an Islamic approach [20, 22].

Additionally, in the present study, for cultural adaptation process the spirituality package was developed by the third author according to Richard and Bergin [22], Ghahari [20] and based on Iranian culture, and it was experimentally implemented on five pregnant women. After addressing the issues and incorporating the suggestions of specialists (five clinical psychology experts, a nursing psychology PhD, and a psychiatrist), the final version was prepared and executed on the experimental group.

Moreover, the intervention is grounded in psychological theories, particularly the Transactional Model of Stress and Coping (TMSC), which emphasizes cognitive appraisal and coping strategies in managing stress. The TMSC is a psychological framework that explains how individuals perceive, interpret, and respond to stressors in their environment. Unlike earlier models that viewed stress as a direct response to external events, the TMSC emphasizes the dynamic interaction between a person and their environment, highlighting the role of cognitive appraisal and coping strategies in shaping stress experiences. This model emphasizes that when individuals encounter or anticipate a stressful event, they engage in a two‐stage appraisal process. First, they evaluate the perceived threat or distress of the situation (primary appraisal), and second, they assess their ability to cope with it (secondary appraisal). Thus, within this model, one aspect of appraisal focuses specifically on coping resources—for instance, spiritual coping where an individual might say “God will help me through this,” while another aspect relates to practical coping strategies [24].

The intervention also integrates forgiveness and gratitude techniques, which are core elements of positive psychotherapy.

The first session focused on introducing the spiritual–religious intervention. In this session, discussions included introducing and justifying the course and its objectives, and educating on pregnancy and childbirth‐related issues for expectant mothers.

The second session focused on discussing *Zekr* (the repetition of holy words) and its connection to mental peace and practicing group meditation. This session covered training on concentration, mindfulness, and spiritual visualization/muscle progressive relaxation.

The third session focused on *Doa* (prayer), where participants were asked to read a short prayer. The fourth session was dedicated to *Tavakkol* (trust in God), during which participants were introduced to the steps of *Tavakkol* and its impact on mental peace. The fifth session covered *Sabr* (patience), exploring its different types and its connection to mental peace. The sixth and seventh sessions addressed *AFV* (forgiveness of self and others). The eighth session was devoted to reviewing the previous sessions. In each session, participants were encouraged to share their related experiences with the group and complete relevant assignments between sessions.

After each session, participants were asked to record their assigned tasks, activities, and thoughts, and provide written reports upon completion of counseling and specific session‐based education (Table 2).

**TABLE 2:** Content of spirituality‐centered counseling sessions.

The sessions	Goals	The topics
First	Introduction and justification of the course and objectives of spiritual care sessions and familiarity with spiritual–religious intervention.	– Introduction and justification of the course and objectives of spiritual care sessions.
		– Examination of life's purpose and meaning from members' perspectives, identification of elements of a meaningful spiritual life, and the effects of finding life's purpose and meaning.
		– Education on issues related to pregnancy and childbirth, including physical changes, understanding the anatomy of the female reproductive system, the process of implantation and growth of the fertilized egg, stages of fetal development, signs of labor onset, description of the contraction process, and stages of natural childbirth.
Second	Awareness of members on how to meditate and focus on various issues, instruction of scientific methods of meditation, practicing meditation in the group, and discussion of Zekr.	Instruction on concentration, meditation, and spiritual visualization/progressive muscle relaxation: visualization and progressive relaxation employing meditation relaxation techniques, accompanied by listening to soothing music (sounds of nature and rain), discussion of the effects of meditation, and presentation of practical methods for meditation.
		Discussion of Zekr (holy words repetition) and its relationship with mental peace.
Third	Improving personal and spiritual communication, achieving inner peace, facilitating the expression of spiritual emotions, and focusing on Doa (prayer).	– Instruction on prayer, supplication, and communication with God; psychological analysis of prayer and supplication and their effects on an individual's relationship with themselves and others.
		– Examination of members' views on prayer and supplication and their experiences in this context.
		– Focus on Doa (prayer) and reading a short prayer by participants.
Fourth	Familiarity with the genuine concept of reliance on God (Tavakkol), including its proper application and timing, and awareness of the role of reliance on God in mental health and inner peace.	– Instruction on reliance on God (Tavakkol), its degrees and types, characteristics of a person who relies on God, the effects of Tavakkol, its role in mental health, and scientific methods of practicing Tavakkol.
		– Implementation of the activity “Daily Patience Performance Worksheet.”
		– Understanding the steps of Tavakkol and its impact on mental peace.
Fifth	Acquaintance with the genuine concept of Sabr (patience), including its proper application and timing, familiarity with Sabr types, and awareness of the role of patience in mental health and inner peace.	– Instruction on patience, its effects, and its impact on mental health, as well as methods to enhance patience.
		– Discussion on the types of Sabr (patience) and its connection to mental peace.
		– Implementation of the activity “Daily Patience Performance Worksheet.”
Sixth	Familiarizing with the correct concept of forgiveness and its distinction from unreasonable forgiveness and awareness of the highly positive impact of forgiveness on mental health.	– Instruction on the skill of forgiveness, including an explanation of the types of forgiveness (forgiving oneself and others, and accepting forgiveness), the stages of forgiveness, and discussion on the roots of lack of forgiveness, identifying the connection between not forgiving oneself and others.
		– Implementation of the activity “Writing a Forgiveness Letter.”
Seventh	Raising awareness of the highly positive impact of gratitude and appreciation on mental health and the reduction of depression.	– Instruction on the skill of gratitude, including the types and positive effects of gratitude, and completion of a gratitude assessment questionnaire.
		– Completion of daily gratitude notebooks.
Eighth	Familiarization with the positive effects of learning the skill of compassion for oneself and others on mental health, inner peace, and reduction of depression, and reviewing the last sessions.	– Instruction on the skill of compassion for oneself and others, including its components, the importance of compassion, and its connection to mental health, altruism, and resilience.
		– Completion of the “Daily Compassion Performance Worksheet” for oneself and others.
		– Reviewing the last sessions.

The intervention was administered by the first and third authors. The group was led by a PhD in Clinical Psychology (third author). Before the intervention, facilitator participated in the workshops and courses of spirituality‐based counseling.

To ensure sessions adherence, in every session, participants were asked to share their related experiences with the group and complete relevant assignments between sessions.

### 2.4 Data Collection

The data collection tools included a demographic and midwifery information form and the Wijma Delivery Expectancy/Experience Questionnaire Version A (W‐DEQ‐A). These tools were administered to participants in three groups at 3 time points: prior to the intervention, 1 week following its completion, and 5 weeks post‐intervention.

### 2.5 The Wijma Birth Expectancy/Experience Scale

The Wijma Delivery Expectancy/Experience Questionnaire Version A (W‐DEQ‐A) comprises 33 items, each rated on a Likert scale ranging from 0 to 5, yielding a total score between 0 and 165. Items 2, 3, 6, 7, 8, 11, 12, 15, 19, 20, 24, 25, 27, and 31 are reverse‐scored. Based on total scores, levels of childbirth fear are categorized as follows: scores ≤ 37 indicate mild fear, 38–65 indicate moderate fear, 66–84 indicate severe fear, and scores ≥ 85 reflect clinical levels of fear. The validity of the W‐DEQ‐A in the Iranian context was confirmed by Andaroon et al. [25]

In this study, the reliability of the questionnaire was assessed using the split‐half method and Cronbach's alpha, resulting in values of 0.71 and 0.83, respectively.

### 2.6 Data Analysis

Statistical analyses were performed using version 25.0 of the Statistical Package for the Social Sciences (SPSS).

The Kolmogorov–Smirnov test was used to assess the normality of quantitative variable distributions. A distribution was deemed normal if the significance level exceeded the critical value of 0.05. For quantitative variables that followed a normal distribution across all three groups, a one‐way analysis of variance (ANOVA) was applied to compare their means. If the data did not meet the normality assumption, the non‐parametric equivalent, the Kruskal–Wallis test, was utilized.

The Kruskal–Wallis test was employed to examine the homogeneity of demographic variables such as age, spouse's age, marital age, and gestational age among the three groups. For continuous variables such as body mass index (BMI) and income, homogeneity was assessed using ANOVA. Categorical demographic variables, including education level, spouse's education, occupation, spouse's occupation, housing status, insurance status, pregnancy intention, fetal gender, spouse support, and support from family and friends, were analyzed using the Chi‐square test. A *p*‐value of less than 0.05 was considered statistically significant for all tests.

The Kruskal–Wallis test was used to determine the difference among the three groups in overall FOC score before the intervention. In addition, the ANOVA test was used to determine the difference among the three groups in overall FOC score 1 week and 5 weeks after the intervention. The Least Square Difference (LSD) post hoc test was used to determine the difference between the groups in overall FOC score 1 week and 5 weeks after the intervention.

We took the assumptions into account for the tests we conducted. For example, in the Chi‐square test, it was required that less than 20% of the cells have an expected value of less than 5, and no cell should have an expected value of zero. In the ANOVA, the assumptions included the normality of continuous variables, the independence of groups, the examination of outlier data through box plots, and the homogeneity of variances in the dependent variables across the independent groups.

The Bonferroni adjustment was applied to determine significance levels for group comparisons [26].

### 2.7 Ethics and Consent

Participants were fully informed about the objectives of the study and provided written informed consent prior to enrollment. They were assured of the confidentiality of their information and informed that participation was voluntary, with the right to withdraw from the study at any time without consequence. Ethical approval was obtained from the Ethics Committee of Kermanshah University of Medical Sciences (Approval Code: IR.KUMS.REC.1401.345). Participants who scored 85 or higher on the W‐DEQ, indicating clinical levels of FOC, were referred to a clinical psychologist at designated healthcare centers. All authors provided consent for the publication of this study in your journal.

## 3 Results

**FIGURE 1:**
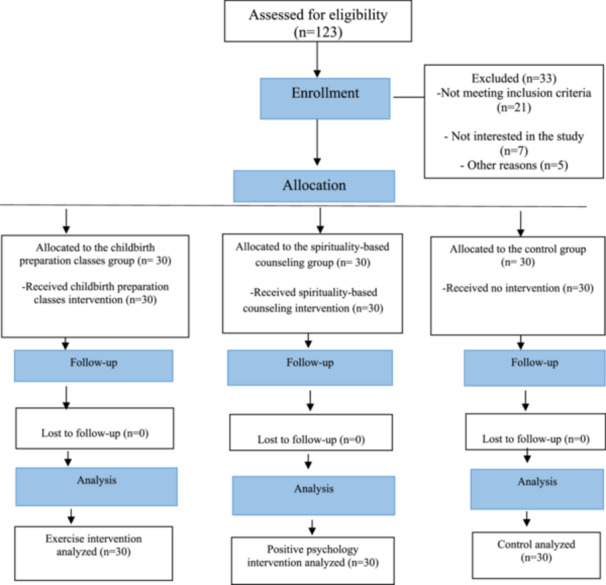
Flowchart of the study.

A total of 123 participants were assessed for eligibility to participate in the study, of whom 90 were deemed eligible and included. In total, 30 participants were assigned to the childbirth preparation classes group, 30 to the spirituality‐based counseling group, and 30 to the control group. No participants were excluded during the follow‐up, and the final number of participants included in the statistical analysis was 90 (Figure 1).

The characteristics of the participants, along with the results of our comparative statistical analyses, are presented in Table 3. No significant differences were found in the individual characteristics among the three groups (*p* > 0.05).

**TABLE 3:** Individual characteristics of pregnant women in the three groups.

Variables		Childbirth preparation classes	Spirituality‐based counseling	Control	*p*‐ value	
		*N* = 30	*N* = 30	*N* = 30		
		Mean ± SD	Mean ± SD	Mean ± SD		
		Or *N* (%)	Or *N* (%)	Or *N* (%)		
Spouse age (year)		33.37 ± 5.59	33.73 ± 6.39	34.80 ± 5.83	0.74*	
Marital age (year)		26.23 ± 5.17	25.47 ± 5.38	27.10 ± 5.55	0.63*	
Gestational age (week)		21.96 ± 1.27	22.00 ± 1.46	21.80 ± 1.18	0.80*	
Body mass index (cm/m²)		23.73 ± 2.15	23.68 ± 2.12	23.67 ± 2.33	*p* > 0.99**	
Income monthly (Toman)		13.53 ± 2.14	13.56 ± 2.86	12.96 ± 3.47	0.66**	
Education level	Lower diploma or Diploma	11 (36.7)	17 (56.7)	12 (40.0)	Total	0.25***
					40 (44.4)	
	University education	19 (66.3)	13 (43.3)	18 (60.0)	50 (55.6)	
Spouse's education	Lower diploma or Diploma	14 (46.7)	17 (56.7)	14 (46.7)	45 (50.0)	0.67***
	University education	16 (53.3)	13 (43.3)	16 (53.3)	45 (50.0)	
Occupation	Employed	7 (23.3)	7 (23.3)	11 (36.76)	25 (27.8)	0.41***
	Housewife	23 (76.7)	23 (76.7)	19 (63.3)	65 (72.2)	
Spouse's occupation	Employed	9 (30.0)	11 (36.7)	12 (40.0)	32 (35.6)	0.71***
	Freelancer	21 (70.0)	19 (63.3)	18 (60.0)	58 (64.4)	
Housing status	Personal	12 (40.0)	7 (23.3)	12 (40.0)	31 (34.4)	0.38***
	Rental	13 (43.3)	15 (50.0)	15 (50.0)	43 (47.8)	
	With family	5 (16.7)	8 (26.7)	3 (10.0)	16 (17.8)	
Insurance status	Yes	17 (56.7)	15 (50.0)	18 (60.0)	50 (55.6)	0.73***
	No	13 (43.3)	15 (50.0)	12 (40.0)	40 (44.4)	
Pregnancy intention	Wanted	16 (53.3)	19 (63.3)	22 (73.3)	57 (63.3)	0.27***
	Unwanted	14 (46.7)	11 (36.7)	8 (26.7)	33 (36.7)	
The fetus gender	Female	18 (60.0)	11 (36.7)	14 (46.7)	43 (47.8)	0.19***
	Male	12 (40.0)	19 (63.3)	16 (53.3)	47 (52.2)	
Spouse support	Yes	23 (76.7)	24 (80.0)	26 (86.7)	73 (81.1)	0.60***
	No	7 (23.3)	6 (20.0)	4 (13.3)	17 (18.9)	
Family and friends support	Yes	23 (76.7)	24 (80.0)	24 (80.0)	71 (78.9)	0.93***
	No	7 (23.3)	6 (20.0)	6 (20.0)	19 (21.1)	

* Kruskal–Wallis test.

** ANOVA test.

*** Chi‐square test.

The Kruskal–Wallis test revealed no significant differences in the mean FOC scores among the three groups before the intervention. However, 1 and 5 weeks post‐intervention, ANOVA identified a significant difference in mean scores across the groups (*p* < 0.05). To further examine these differences, the LSD post hoc test was used to compare the overall FOC scores 1 week after the intervention. A significant difference was found between the Spirituality‐based counseling group and the control group (*p* < 0.001) and between the Childbirth preparation classes group and the control group (*p* < 0.001). No significant difference was found between the Childbirth preparation classes and Spirituality‐based counseling groups (Table 4).

**TABLE 4:** Comparison of the mean and standard deviation of the total score of fear of childbirth between three groups.

Times	Childbirth preparation classes	Spirituality‐based counseling	Control group	Statistical value	Bonferroni‐adjusted *p*‐value
	Mean ± SD	Mean ± SD	Mean ± SD		
Before the intervention	50.97 ± 4.75	53.23 ± 4.87	53.03 ± 5.12	3.64	0.48*
1 week after the intervention	40.20 ± 4.97	43.06 ± 10.14	54.66 ± 5.60	33.21	< 0.001** ^,^ a ^,^ b ^,^ c
5 weeks after the intervention	35.26 ± 4.20	30.50 ± 6.13	61.60 ± 5.26	304.02	< 0.001** ^,^ d ^,^ e ^,^ f
*p*‐value	< 0.001****	< 0.001****	< 0.001***		

* Kruskal–Wallis test.

** ANOVA test.

*** Friedman test.

**** Repeated measure test.

^a^ A significant statistical difference was seen between the Spirituality‐based counseling and control group (*p* < 0.001) by the Least Significant Difference post hoc test.

^b^ A significant statistical difference was seen between the Childbirth preparation classes and control group (*p* < 0.001) by the Least Significant Difference.

^C^ A significant statistical difference was not seen between the Childbirth preparation classes and the Spirituality‐based group (*p* = 0.131) by the Least Significant Difference.

^d^ A significant statistical difference was seen between the Spirituality‐based counseling and control group (*p* < 0.001) by the Least Significant Difference.

^e^ A significant statistical difference was seen between the Childbirth preparation classes and the control group ( < 0.001) by the Least Significant Difference.

^f^ A significant statistical difference was seen between the Childbirth preparation classes and the Spirituality‐based group (*p* = 0.001) by the Least Significant Difference.

The LSD post hoc test was also applied to assess differences in FOC scores 5 weeks after the intervention. A statistically significant difference was found between the Control and Spirituality‐based counseling groups (*p* < 0.001) and between the Childbirth preparation classes and Control groups (*p* < 0.001). Additionally, a statistically significant difference was observed between the Childbirth preparation classes and Spirituality‐based counseling groups (*p* = 0.001) (Table 4).

According to the findings of the Friedman test, or repeated measure test the trend of changes in the mean childbirth fear score was significant in three groups (*p* < 0.05) (Table 4).

A Bonferroni adjustment method used to control for Type I errors (Table 4).

Ultimately, the variables that had a *p*‐value equal to or less than 0.2 in the univariate analysis were entered into the regression model using the backward method (Table 5). As shown in the Table 5, the value of the non‐standard coefficient (*B*) is equal to 1.023 that indicates a one‐unit increase in the pre‐intervention fear score adds 1.023 to the total fear score 1‐week post‐intervention. Additionally, a one‐unit increase in the pre‐intervention fear score adds 0.834 to the total fear score 5 weeks post‐intervention (Table 6).

**TABLE 5:** The results of the regression Model.

	Non‐standard coefficient		Standard coefficient	*t*	Sig.
	*B*	Std. error	Beta		
(Constant)	−7.648	9.129		−0.838	0.404
Pre‐intervention fear score	1.023	0.173	0.532	5.900	< 0.001

*Note:* Dependent variable: total fear score 1‐week post‐intervention. Independent variables: pre‐intervention fear score and infant sex.

**TABLE 6:** The results of the regression model.

	*B*	Std. error	Beta	*t*	Sig.
(Constant)	−1.276	15.916		−0.080	0.936
Pre‐intervention fear score	0.834	0.302	0.282	2.760	0.007

*Note:* Dependent variable: total fear score 5 weeks post‐intervention. Independent variables: pre‐intervention fear score and infant sex.

As can be seen, the results indicate that the effect of the intervention on FOC was statistically significant and the baby's gender variable which was significant in univariate analysis did not remain significant in regression, but fear was still significant. Therefore, the effect of the intervention on fear was not influenced by the infant gender.

A similar result was obtained in the analysis of covariance (ANCOVA), where the pre‐intervention fear score was considered as a covariate. The results indicated that the pre‐intervention fear score was significantly associated with the fear scores 1 week and 5 weeks post‐intervention.

## 4 Discussion

The present study aimed to compare the effects of childbirth preparation classes and spirituality‐based counseling on FOC in primiparous pregnant women. The results of the study indicated that 1 week after the intervention, both childbirth preparation classes and spirituality‐based counseling equally reduced FOC. However, 5 weeks after the intervention, spirituality‐based counseling was more effective in reducing the FOC compared to childbirth preparation classes. A review of the literature revealed an absence of studies that examined the same interventions as those investigated in the current research. Therefore, this section compares the results of the present study with the findings from related studies. Consistent with the results of the present study, Hasanzadeh et al. found that the scores for FOC were significantly lower in pregnant women who regularly attended childbirth preparation classes compared to pregnant women who did not attend these classes [27]. A quasi‐experimental study conducted in Turkey showed that the intervention group (antenatal education) had a lower mean score for FOC compared to the control group [28]. Another study concluded that childbirth preparation classes + childbirth fear coping techniques such as breathing exercises and relaxation techniques in addition to routine antenatal care have a significant effect on childbirth fears in primipara women compared to those who received only routine antenatal care (control group) and the mean score related childbirth fears of the study group after the intervention was lower than the mean score of the control group [29]. Kacperczyk‐Bartnik et al. found that the primiparas women who attended antenatal classes scored lower on the Delivery Fear Scale [30]. In a prospective cohort study 202 primiparous pregnant women divided into two groups of willing and unwilling to attend childbirth preparation classes, a significant difference was observed in fear scores between the two groups in the third trimester and after attending classes, as the mean score of FOC was lower in the attending group than that in the routine care group after the end of the classes [31]. In a study conducted by Toohill et al. on 339 pregnant women with severe fear, telephone counseling resulted in a greater reduction in FOC scores compared to no intervention [32]. It appears that participation in childbirth preparation classes reduces FOC by increasing mothers' knowledge about pregnancy, childbirth, and the postpartum period, and by boosting their confidence in their ability to endure labor pain [33, 34]. Additionally, in childbirth preparation classes, women can utilize breathing techniques, physical exercises, and relaxation techniques, which help reduce fear and stress related to childbirth.

Furthermore, some studies suggest that greater spiritual intelligence in pregnant women may alleviate their FOC [35]. Additionally, other research has identified a significant negative relationship between spirituality and FOC, indicating that higher levels of spirituality can encourage women to opt for natural childbirth over cesarean delivery, thus promoting a safer childbirth experience [36]. The study by Rabiepour and Etesami found a significant association between spiritual well‐being and FOC in pregnant women, indicating that greater spiritual well‐being is linked to reduced FOC [37]. A study by Biglich et al., which aimed to analyze the relationship between FOC and spiritual well‐being, revealed a negative correlation between the two variables. Specifically, higher levels of spiritual well‐being were associated with an 18% reduction in FOC [38]. Similarly, the findings of a study by Akbarzadeh et al. demonstrated that spirituality during pregnancy can enhance the sense of control and foster psychological adaptability to the stresses of pregnancy and childbirth. The belief in an omnipotent God, who is present in all situations and observes His creations, can significantly alleviate anxieties related to the situation. In other words, individuals may feel able to manage the uncontrollable by relying on God [39]. These findings align with the results of our study, highlighting that the primary and most significant effect of spirituality in managing FOC is its influence on an individual's attitude and interpretation of pregnancy. Pregnant women utilize various resources and strategies to manage stress, and their beliefs about pregnancy and childbirth play a critical role in shaping cognitive evaluations during the process of addressing FOC. Spirituality, in particular, aids pregnant women in reframing and interpreting the painful experience of childbirth more positively and constructively [40].

As mentioned before, 5 weeks after the intervention, spirituality‐based counseling was more effective in reducing the FOC compared to childbirth preparation classes.

As previously mentioned, no study was found that compares the impact of childbirth preparation classes and spirituality‐based counseling on the FOC. However, in the study by Karimi et al., which investigated the effects of cognitive counseling and childbirth preparation classes, the results indicated that immediately after the intervention, there was no significant difference in FOC between the cognitive counseling group and the physiological childbirth education group, 4 weeks post‐intervention, a significant difference in fear levels was observed between the two groups. Cognitive counseling was found to result in a greater reduction in FOC [41]. Results of another study showed that art therapy based on painting was more effective in comparison with childbirth preparation classes in reducing the psychological distress of pregnant women in the third trimester [42].

It appears that spirituality‐based counseling helps women better understand their spiritual needs during pregnancy and childbirth. With this understanding, they can make more informed decisions regarding themselves and their childbirth process. This type of counseling typically includes attention to the beliefs, values, and spiritual needs of women and may suggest strategies for improving physical, emotional, and psychological functioning. Furthermore, higher levels of spiritual well‐being during pregnancy can serve as a protective factor, providing support throughout this period. Spirituality and a connection with a higher power have been shown to reduce stress and anxiety while enhancing psychological well‐being and emotional stability [43]. In a study conducted by Naranji et al., unemployed women who engaged in religious practices such as performing Namaz (prayers), worshiping, and strengthening their relationship with God reported lower levels of anxiety and fear, thereby facilitating a more positive experience during this period [44]. Religious beliefs and practices can strengthen an individual's connection to their inner spirituality. Among pregnant women, practices such as maintaining a relationship with God through prayer have been widely observed. Moreover, individuals who perceive God as a source of security and inner refuge tend to exhibit greater resilience when confronting challenges [45]. Khodabakhshi et al. demonstrated that pregnant women with higher levels of spiritual well‐being exhibit greater resilience in coping with stress and anxiety, and are more likely to have a natural delivery [46]. Similarly, McCasker et al. found that individuals with elevated spiritual well‐being display greater adaptability when confronted with novel and uncertain situations, such as natural childbirth. Furthermore, these individuals are better equipped to manage stress and mitigate its negative effects [47]. Hatami et al., in their analysis of the relationship between spiritual well‐being and resilience with a focus on FOC in pregnant women, concluded that spiritual well‐being empowers women to confront the challenges of natural childbirth. This is achieved by enhancing their self‐control and efficacy through concepts such as finding meaning and purpose in life, maintaining hope for the future, and seeking support from a higher power to navigate this demanding experience. Accordingly, the undesirable psychological, emotional, physical, and economic consequences of unnecessary cesarean sections, particularly those driven by psychological factors, can be minimized [21].

Furthermore, spirituality has deep roots in Iran, and the influence of spiritual beliefs is evident in all aspects of Iranian society. Therefore, it can be said that spirituality‐based interventions are well aligned with existing cultural practices in Iran.

The present study showed that in the control group, not only did the levels of FOC not decrease over time, but they increased progressively with the advancement of pregnancy. These findings are consistent with the results of other studies [27, 45]. It seems that due to the lack of adequate information in the control group, women had less access to resources and education about childbirth, methods to reduce fear, and their abilities to cope with these challenges. Consequently, their fear increased over time. In contrast, the intervention groups typically used various methods to facilitate childbirth, such as physical exercises, breathing techniques, and mental and psychological strategies. These methods help individuals gain more control over their bodies during childbirth and feel more secure about the process. The absence of these methods in the control group could lead to an increase in FOC [8, 27, 48, 49].

## 5 Strengths and Limitations

### 5.1 Strengths

The study utilized standardized tools, including the Wijma Delivery Expectancy/Experience Questionnaire (W‐DEQ) for assessing FOC. The interventions were based on established protocols, specifically the national protocol from the Ministry of Health, Treatment, and Medical Education regarding childbirth preparation classes and spirituality‐based counseling.

### 5.2 Limitations

Our findings are specific to primiparous women from a particular region and cultural context. Future research involving more diverse populations, including multiparous women and settings outside Iran, with longer‐term follow‐up to assess the durability and broader applicability of the interventions are recommended. The non‐randomized, quasi‐experimental design exposing the study to selection bias and compromising its internal validity. However, we employed careful matching of participants across groups based on key demographic variables to minimize potential selection bias.

Moreover, the control group received only routine care, whereas both intervention groups were afforded substantial additional contact time and structured sessions, an imbalance that could itself account for changes in fear levels rather than the specific content of the interventions. In future studies, including an attention‐control group to better isolate the specific effects of the intervention content is recommended.

Additionally, only women who scored between 38 and 84 on the W‐DEQ were included in the study, which might reduce the generalizability study indicate that spirituality‐based counseling leads to a reduction in women's FOC 1 week and 5 weeks after the end of the intervention, with a more significant reduction observed 5 weeks post‐intervention. Additionally, participation in childbirth preparation classes resulted in decreased FOC, although its impact was less compared to spirituality‐based counseling. Therefore, it is recommended that the current protocol of childbirth preparation classes, which provides general education to prepare women for safe and physiological childbirth, be reviewed nationwide. By incorporating spiritual counseling programs into prenatal care plans, effective utilization of its outcomes for the improvement of women's and neonates' health can be achieved.

## Author Contributions

**Fatemeh Golnazari:** methodology, resources, writing – review and editing. **Sousan Heydarpour:** conceptualization, methodology, supervision, writing – review and editing, project administration. **Aliakbar Foroughi:** methodology, validation. **Fatemeh Heydarpour:** formal analysis, methodology, validation, data curation.

## Disclosure

The lead author Sousan Heydarpour affirms that this manuscript is an honest, accurate, and transparent account of the study being reported; that no important aspects of the study have been omitted; and that any discrepancies from the study as planned (and, if relevant, registered) have been explained.

## Consent

The authors have nothing to report.

## Conflicts of Interest

The authors declare no conflicts of interest.

## Data Availability

The data that support the findings of this study are available from the corresponding author upon reasonable request.
